# A trawl collected dataset of *Johnius* (Actinopterygii, Sciaenidae) species in central-western Taiwanese waters

**DOI:** 10.3897/BDJ.12.e117960

**Published:** 2024-06-27

**Authors:** Norhafiz Hanafi, Meng-Hsien Chen, Ming-Che Yang, Chien-Cheng Lai, Chih-Wei Chang

**Affiliations:** 1 Department of Oceanography, National Sun Yat-sen University (NSYSU), Kaohsiung City 80424, Taiwan Department of Oceanography, National Sun Yat-sen University (NSYSU) Kaohsiung City 80424 Taiwan; 2 Institute of Marine Ecology and Conservation, National Sun Yat-sen University (NSYSU), Kaohsiung City 80424, Taiwan Institute of Marine Ecology and Conservation, National Sun Yat-sen University (NSYSU) Kaohsiung City 80424 Taiwan; 3 Marine Ecology and Conservation Research Center, National Academy of Marine Research, Kaohsiung 80661, Taiwan., Kaohsiung, Taiwan Marine Ecology and Conservation Research Center, National Academy of Marine Research, Kaohsiung 80661, Taiwan. Kaohsiung Taiwan; 4 National Museum of Marine Biology and Aquarium, and Graduate Institute of Marine Biology, National Dong Hwa University, Pingtung 944, Taiwan, Pingtung, Taiwan National Museum of Marine Biology and Aquarium, and Graduate Institute of Marine Biology, National Dong Hwa University, Pingtung 944, Taiwan Pingtung Taiwan

**Keywords:** croacker, abundance, Chang-yun Rise, spatial distribution, 2°C water temperature difference

## Abstract

**Background:**

Sciaenidae is one of the most important coastal fisheries in Taiwan, both in production and economic value. It is also significant as the main targetted diet of Chinese white dolphins, *Sousachinensis*, especially for the genus *Johnius*, such as *J.taiwanensis*, *J.belangerii* and *J.distinctus*, which is primarily found in central-western Taiwan coastal waters. Despite an abundance of *Johnius* species occurrences reported in the Global Biodiversity Information Facility (GBIF) and the Taiwan Biodiversity Information Facility (TaiBIF) data portals (Mozambique, Australia, Taiwan, Korea, India, Indonesia, South Africa, Pakistan, Vietnam and China), there are no specific datasets that properly document the regional distribution of this genus, especially in Taiwanese waters. Thus, this paper describes a dataset of genus *Johnius* occurrences in waters on the central-western coast of Taiwan. The data collection for the present study was conducted from 2009 until 2020 and comprised 62 sampling events and 133 occurrence records. All fish specimens were collected by trawling in Miaoli, Changhwa and Yunlin Counties, Taiwan and brought back to the lab for identification, individual number count and body weight measurement. These processing data have been integrated and established in the Taiwan Fish Database and published in GBIF. This dataset contains six *Johnius* species and 2,566 specimens, making it comprehensive *Johnius* fish fauna and spatial distributional data on the coastal habitat in central-western Taiwanese waters.

**New information:**

This dataset contains 133 occurrence records of *Johnius* species (Sciaenidae) with 2,566 specimens, making it the most extensive public dataset of *Johnius* distribution records in Taiwan. The publication of this dataset through the TaiBIF and GBIF dataset platforms demonstrated that the number of *Johnius* spatial and temporal records in Taiwan waters is influenced by the topographical structure of the Changyun Rise (CYR) in combination with the cold current of the China Coastal currents and bound with the warm currents of the Kuroshio and the South China Sea on the central-western coast of Taiwan. The data serve as the foundation for understanding the biogeography and *Johnius* species ecology in Taiwan's coastal waters, which present a 2°C water temperature difference split at the CYR.

## Introduction

*Johnius* are short migratory coastal species that travel less than 100–200 m between estuaries and neritic water during their life cycle (Lin et al. 2007). Fricke et al. (2023) identified 35 legitimate *Johnius* species found in the Indo-West Pacific area and classified them into two subgenera: Johnius (Johnius) and Johnius (Johnieops), the latter distinguished by a row of larger teeth on the lower jaw (Lal Mohan 1975). Amongst the Indo-West Pacific Sciaenidae, *Johnius* is the largest and most taxonomically perplexing genus. Moreover, *Johnius* has remarkable diversity and is a global species, the monophyly of which has been established (Trewavas 1977, Lo et al. 2015, Lo et al. 2017). In Taiwan and Southeast Asia particularly, several studies have been conducted on the reproduction ecology and biological organism of *Johnius* (Jan et al. 2006, Lin et al. 2007, Mok et al. 2009, Jan et al. 2010, Zhang et al. 2019), owing to the significant accessibility of the dispersal and migration between spawning sites. Nonetheless, understanding of their abundance, diversity and ecological function is still limited in this region.

Recognising this gap, this study initiative and extent to collect the "*Johnius* (croaker)" specimens focuses on the occurrences of *Johnius* species in order to understand their distribution and abundance in western coastal Taiwan. This project ran from 2009 until 2020 and collected an abundance of samples at several sites (Miaoli, Changhwa and Yunlin) along the coastline of western Taiwan. In addition to documenting occurrence reports, this study aimed to develop a comprehensive dataset of *Johnius* species profiles in central-western Taiwan. By employing morphological identification methods for individual fish, we sought to elucidate their distribution patterns within the region. Thus, to encourage continuous reports of the occurrence pattern, we built up the distributional pattern in our database.

The purpose of preparing the current dataset was to publicly open the data for advancement, especially in the scientific and conservation communities. The information regarding the *Johnius* genus in Taiwan has facilitated a fundamental comprehension of the biogeography concerning the distribution pattern of *Johnius* species in Taiwan, along with certain ecological factors that influence their distribution and migratory patterns in the waters surrounding Taiwan.

## Sampling methods

### Study extent

All of the *Johnius* samples included in the dataset were obtained by trawling on the central-western coast of Taiwan.

### Sampling description

**Figure 1.**
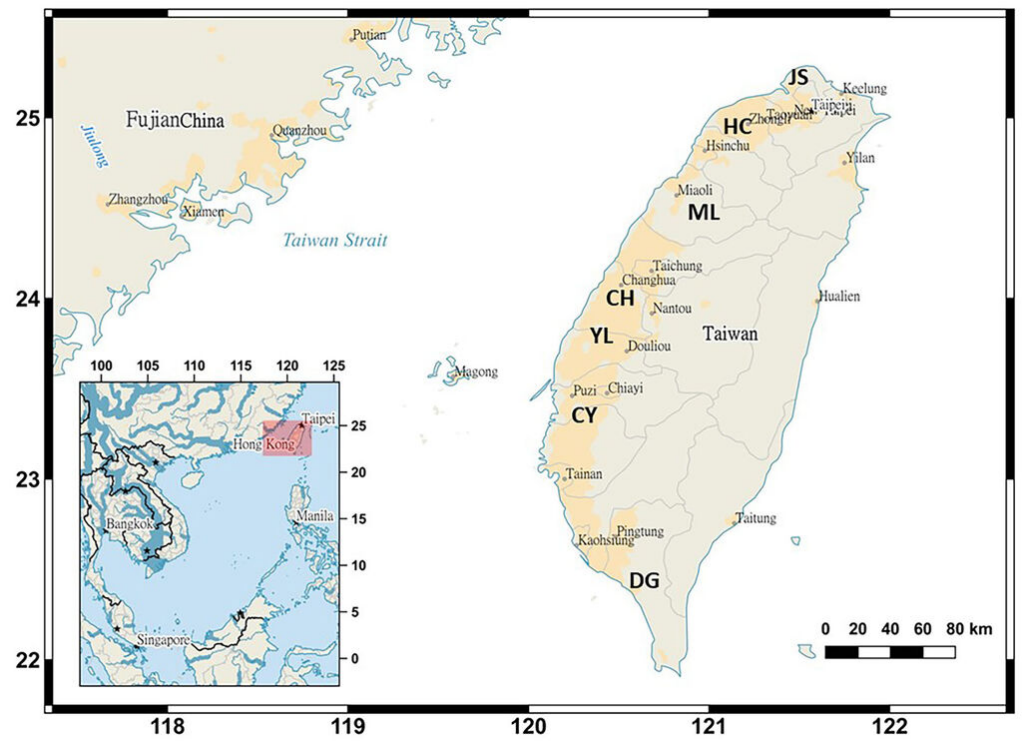
Sampling sites of *Johnius* species in Taiwan (Acronyms for sites: Jinshan (JS), Hsinchu (HC), Miaoli (ML), Changhwa (CH), Yunlin (YL), Chiayi (CY) and Donggang (DG).

The sampling design for collecting bottom-trawl harvest from coastal waters around central-western Taiwan is shown in Fig. 1. The abbreviations of locality names of the sampling sites are outlined below (Fig. 1). The latitude and longitude of trawling routes were plotted on Google Maps and were shown in occurrence datasets in GBIF. Several series of extensive monthly samples collected from Miaoli, Changhwa and Yunlin were analysed starting in 2009, followed by the Yunlin collection from 2012–2016 and Miaoli fish landing ports in north-western Taiwan waters between March 2019 and March 2020 (depth of trawl operation: 100-500 m; distance trawl from shore: 1000 m; mesh size of the net: 10 mm; trawling duration: 30 minutes by vessels). The fresh fish samples were utilised as reference species for identification, based on their morphological characteristics, supported by specimen vouchers (Chao et al. 2019, Hanafi et al. 2023a).

Coordinates of sampling sites: Changhwa: 24.1579°N, 120.3837°E; Miaoli: 24.6179°N, 120.6221°E; and Yunlin: 23.7029°N, 120.1022°E.

### Quality control

All the scientific names of fish samples were validated by the updated fish checklist in the Taiwan Fish Database before being added to the database. Subsequently, the data were re-validated by cross-referencing them with Chao et al. (2019) and Hanafi et al. (2023b) as well as referring to FishBase, Catalogue of Fishes and the California Academy of Sciences for further verification. The latitude and longitude of trawling routes were plotted on Google Maps and were shown in occurrence datasets in GBIF. A total of six specimens had been deposited at the National Museum of Marine Biology and Aquarium, Taiwan (NMMBA) Institution for each of the species as voucher specimens (*Johniusamblycephalus*: NMMBP37066; *J.belangerii*: NMMBP37067; *J.borneensis*: NMMBP37068; *J.distinctus*: NMMBP37069; *J.trewavasae*: NMMBP37071; *J.taiwanensis*: NMMBP37073; and *J.grypotus*: NMMBP23015).

### Step description

Data collection. The integration of *Johnius* distributional data started in 2009 to 2010 in Changhwa County, while data from 2012 to 2016 were acquired from the Master’s dissertation of Liou (2016). Data from both were collected seasonally by trawl fishing. Meanwhile, from March 2019 to March 2020, the samples collected in Miaoli were collected monthly by either trawling in the coastal waters of Taiwan or from fish markets. A total of 2,566 *Johnius* specimens were used to investigate their spatial distribution in Taiwanese waters. After the sample collections had been sorted, all the specimens were identified to the species level (Chao et al. 2019, Hanafi et al. 2023b) and their length (in mm) and weight (in g) were measured.

Open data preparation: We converted the occurrence data into Darwin Core Archive standard in Google Sheets, an online spreadsheet tool, using the Darwin Core Archive Assistant Add-on (Salim and Saraiva 2020). The data were then made available on the IPT TaiBIF and GBIF for the public to access.

## Geographic coverage

### Description

We downloaded the Landsat 8 satellite image on 18 September 2022 and drew the investigation scope by using the ArcGIS 10.7 software. Our survey covered central-western areas of Taiwan Island, such as inshore and offshore coastal waters.

### Coordinates

22.7964 and 25.3441 Latitude; 119.8168 and 121.2451 Longitude.

## Taxonomic coverage

### Description

**Figure 2.**
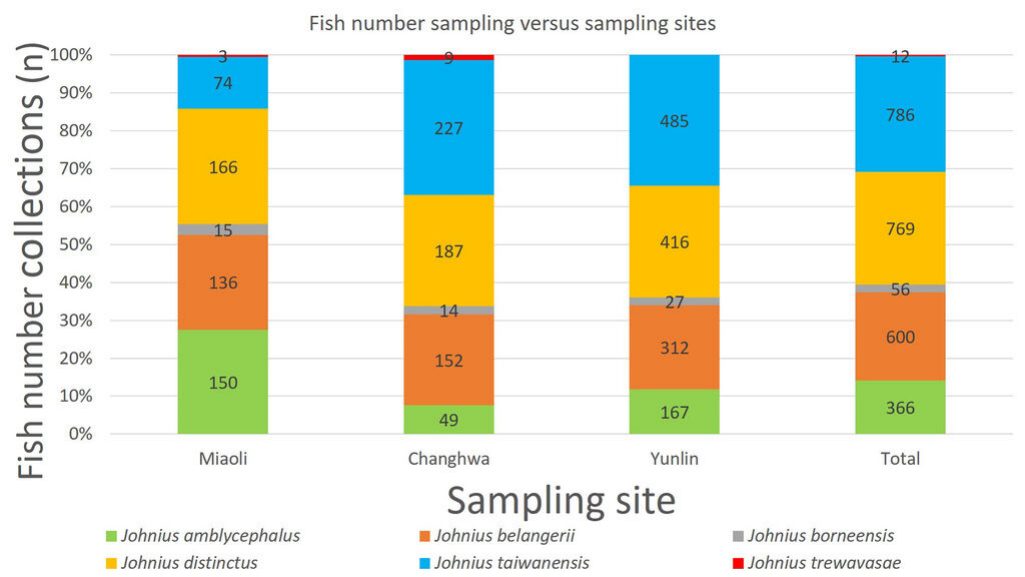
The histogram shows the number of *Johnius* species composition versus sampling site collections covered in this study.

**Figure 3.**
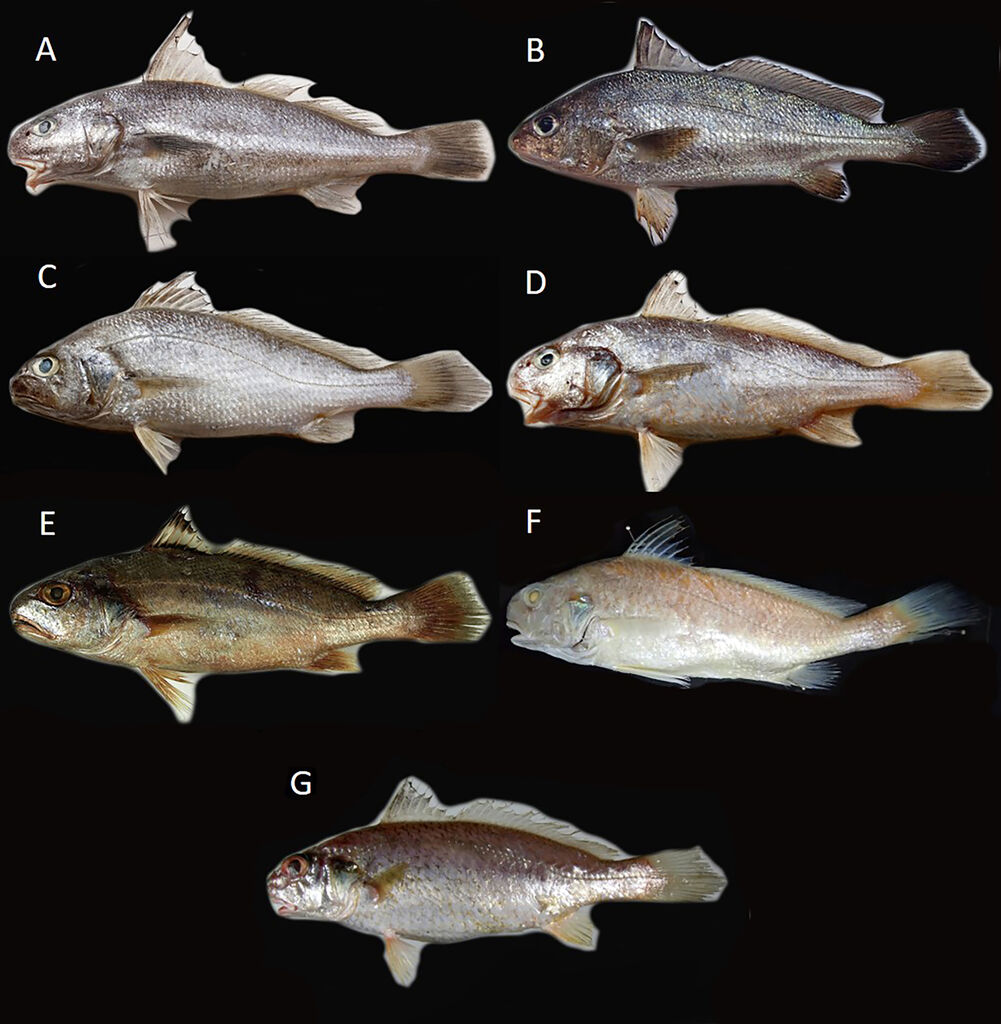
Photographs of the comparative materials: (A) *Johniusamblycephalus* NMMB-P 37066, 137.09 mm SL; (B) *Johniusbelangerii* NMMB-P 37067, 139.37 mm SL; (C) *Johniusborneensis* NMMB-P 37068, 101.86 mm SL; (D) *Johniustaiwanensis* NMMB-P 37073, 122.62 mm SL; (E) *Johniusdistinctus* NMMB-P 37069, 87.27 mm SL; (F) *Johniusgrypotus* NMMB-P 23015, 140.22 mm SL; and (G) *Johniustrewavasae* NMMB-P 37071, 117.42 mm SL.

**Figure 4.**
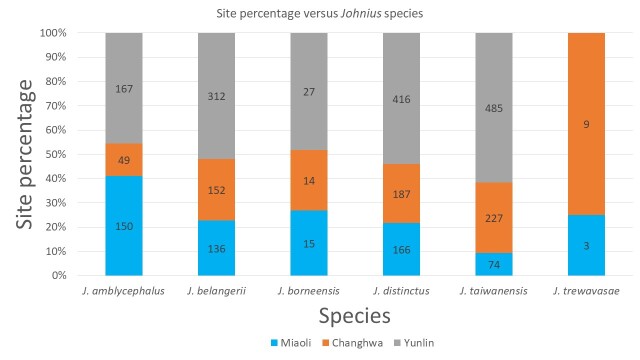
*Johnius* species composition and abundance in each locality in central-western Taiwan.

**Figure 5.**
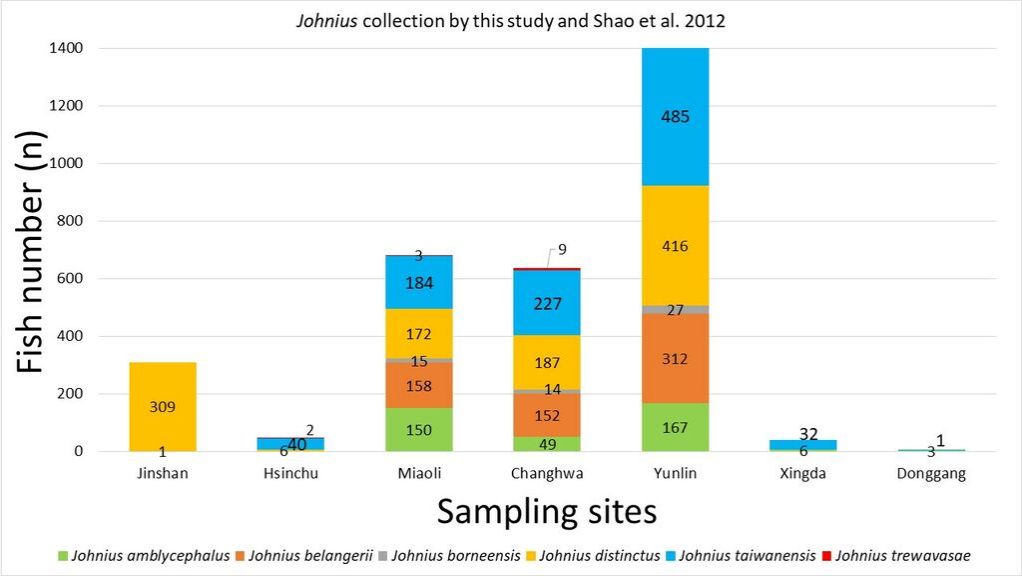
*Johnius* spatial distribution integrated data by this study and Shao et al. (2012) collected by trawling survey in central-western Taiwanese coastal survey.

Six species of *Johnius* were recorded in the dataset (Fig. 2) derived from the collection sites and the composition of *Johnius* species, namely *Johniusamblycephalus* (Bleeker, 1855) - Bearded croaker, *J.belangerii* (Cuvier, 1830) Belanger's croaker, *J.borneensis* (Bleeker, 1850), Sharpnose hammer croaker, *J.distinctus* (Tanaka, 1916), Distinct's croaker, *J.taiwanensis* Chao et al., 2019, Taiwan's croaker and *J.trewavasae* Sasaki, 1992, Trewavas croaker (Fig. 3). Only one sole *sciaenid* species, *J.grypotus* Sasaki, 1990 (Fig. 3), described by Yu and Shen (1987) occurrences in Keelung, northern Taiwan and resurrected by Sasaki (1990), was not found in this study. The most abundant species in the dataset were *J.belangerii*, *J.distinctus* and *J.taiwanensis* (23.2%, 29.7% and 30.4%, respectively) (Fig. 4). A photograph of sample material of *Johnius* species was cited by Hanafi et al. (2023b). Comparison data with the Shao et al. (2012) collection sample are also presented in Fig. 5, as comprehensive information on *Johnius* spatial distribution stretching from northern to southern-western Taiwanese coastal waters was integrated into the present study. A finding of the present study was the occurrence of *J.trewavasae* in Miaoli and Changhwa, which Shao et al. (2012) did not collect in their sampling survey. In addition, the collection data did not acquire any *J.grypotus* occurrences in Taiwanese coastal waters during the sampling survey series.

## Temporal coverage

### Notes

This survey was conducted from the year 2009 in many series collections seasonally from 2009 to 2016 (in Changhwa and Yunlin) and monthly from July 2019 to June 2020 (in Miaoli). The specific dates of the collection are shown in the resources link. All the occurrence information with integration data follow the format of the sampling description.

## Usage licence

### Usage licence

Creative Commons Public Domain Waiver (CC-Zero)

### IP rights notes

Creative Commons Attribution Non Commercial (CC-BY-NC) 4.0 License

## Data resources

### Data package title

A trawling collected dataset of *Johnius* (Actinopterygii, Sciaenidae) species in central-western Taiwanese waters

### Resource link

doi.org/10.15468/cnpayh

### Alternative identifiers

https://ipt.taibif.tw/resource?r=hafizhanafi&v=1.19

### Number of data sets

1

### Data set 1.

#### Data set name

A trawling collected dataset of *Johnius* (Actinopterygii, Sciaenidae) species in central-western Taiwanese waters

#### Data format

Darwin Core Archive format

#### Description

The dataset contains data associated with the sampling event series and occurrence of individual *Johnius* species (Hanafi et al. 2023a), which occur in the central-western Taiwanese waters. The category consists of information during the sampling series event according to the Darwin Core Standard. The data allowed future research studies, such as biogeography and *Johnius* ecology, that include habitat use and intra- and interspecies interaction. The data may also potentially guide any policy-making process through the assessment of species conservation status and diversity in the area of occurrences.

**Data set 1.** 

Column label	Column description
eventID (Event Core, Occurrence Extension)	An identifier for the set of information associated with an Event (something that occurs at a place and time). May be a global unique identifier or an identifier specific to the dataset.
eventDate (Event Core)	The date-time or interval during which an Event occurred. For occurrences, this is the date-time when the event was recorded. Not suitable for a time in a geological context.
locality (Event Core)	The specific description of the place.
samplingProtocol (Event Core)	The names of, references to, or descriptions of the methods or protocols used during a dwc:Event.
year (Event Core)	The four-digit year in which the Event occurred, according to the Common Era Calendar.
month (Event Core)	The integer month in which the Event occurred.
day (Event Core)	The integer day of the month on which the Event occurred.
country (Event Core)	The name of the country or major administrative unit in which the Location occurs.
countryCode (Event Core)	The standard code for the country in which the Location occurs.
decimalLatitude (Event Core)	The geographic latitude (in decimal degrees, using the spatial reference system given in geodeticDatum) of the geographic centre of a Location. Positive values are north of the Equator, negative values are south of it. Legal values lie between -90 and 90, inclusive.
decimalLongitude (Event Core)	The geographic longitude (in decimal degrees, using the spatial reference system given in geodeticDatum) of the geographic centre of a Location. Positive values are east of the Greenwich Meridian, negative values are west of it. Legal values lie between -180 and 180, inclusive.
geodeticDatum (Event Core)	The ellipsoid, geodetic datum or spatial reference system (SRS) upon which the geographic coordinates given in decimalLatitude and decimalLongitude are based.
coordinateUncertaintyInMetres (Event Core)	The horizontal distance (in metres) from the given decimalLatitude and decimalLongitude describing the smallest circle containing the whole of the Location. Leave the value empty if the uncertainty is unknown, cannot be estimated or is not applicable (because there are no coordinates). Zero is not a valid value for this term.
sampleSizeValue (Event Core)	A numeric value for a measurement of the size (time duration, length, area or volume) of a sample in a sampling dwc:Event.
sampleSizeUnit (Event Core)	The unit of measurement of the size (time duration, length, area or volume) of a sample in a sampling dwc:Event.
samplingEffort (Event Core)	The amount of effort expended during a dwc:Event.
occurrenceStatus (Occurrence Extension)	A statement about the presence or absence of a dwc:Taxon at a dcterms:Location.
organismQuantity (Occurrence Extension)	A number or enumeration value for the quantity of dwc:Organisms.
organismQuantityType (Occurrence Extension)	The type of quantification system used for the quantity of dwc:Organisms.
occurrenceID (Occurrence Extension)	An identifier for the dwc:Occurrence (as opposed to a particular digital record of the dwc:Occurrence). In the absence of a persistent global unique identifier, construct one from a combination of identifiers in the record that will most closely make the dwc:occurrenceID globally unique.
recordedBy (Occurrence Extension)	A list (concatenated and separated) of names of people, groups or organisations responsible for recording the original dwc:Occurrence. The primary collector or observer, especially one who applies a personal identifier (dwc:recordNumber), should be listed first.
basisOfRecord (Occurrence Extension)	The specific nature of the data record.
individualCount (Occurrence Extension)	The number of individuals present at the time of the dwc:Occurrence.
taxonID (Occurrence Extension)	A global unique identifier for the taxon (name in a classification).
taxonRank (Occurrence Extension)	The taxonomic rank of the most specific name in the scientificName.
scientificName (Occurrence Extension)	The full scientific name, with authorship and date information if known. When forming part of an Identification, this should be the name in lowest level taxonomic rank that can be determined. This term should not contain identification qualifications, which should instead be supplied in the IdentificationQualifier term.
vernacularName (Occurrence Extension)	A common or vernacular name.
language (Occurrence Extension)	The language used in the dataset.
kingdom (Occurrence Extension)	The full scientific name of the kingdom in which the taxon is classified.
phylum (Occurrence Extension)	The full scientific name of the phylum in which the taxon is classified.
class (Occurrence Extension)	The full scientific name of the class in which the taxon is classified.
order (Occurrence Extension)	The full scientific name of the order in which the taxon is classified.
family (Occurrence Extension)	The full scientific name of the family in which the taxon is classified.
genus (Occurrence Extension)	The full scientific name of the genus in which the taxon is classified.
specificEphitet (Occurrence Extension)	The name of the first or species epithet of the scientificName.
